# FODSeg: a deep learning framework for tract-specific white matter segmentation from full angular distributions

**DOI:** 10.3389/fnins.2025.1734498

**Published:** 2026-01-12

**Authors:** Ankita Joshi, Hailong Li, Nehal A. Parikh, Lili He

**Affiliations:** 1 Department of Radiology, Imaging Research Center, Cincinnati Children’s Hospital Medical Center, Cincinnati, OH, United States; 2 Neurodevelopmental Disorders Prevention Center, Perinatal Institute, Cincinnati Children’s Hospital Medical Center, Cincinnati, OH, United States; 3 Artificial Intelligence Imaging Research Center, Cincinnati Children’s Hospital Medical Center, Cincinnati, OH, United States; 4 Department of Radiology, University of Cincinnati College of Medicine, Cincinnati, OH, United States; 5 Department of Pediatrics, University of Cincinnati College of Medicine, Cincinnati, OH, United States; 6 Department of Computer Science, University of Cincinnati, Cincinnati, OH, United States; 7 Department of Biomedical Engineering, University of Cincinnati, Cincinnati, OH, United States; 8 Department of Biomedical Informatics, University of Cincinnati, Cincinnati, OH, United States

**Keywords:** bottleneck issues, crossing fibers, deep learning, diffusion magnetic resonance imaging, tractography, white matter tract segmentation

## Abstract

**Introduction:**

White matter tract segmentation is critical for mapping brain connectivity in both clinical and research settings. Recent deep learning methods have enabled direct voxel-wise segmentation from diffusion MRI (dMRI), bypassing tractography. However, most approaches rely on a limited number of peaks extracted from the fiber orientation distribution function (fODF) at each voxel, which discards important orientation information, particularly in problematic regions with complex fiber configurations such as crossing fibers and bottlenecks.

**Methods:**

In this work, we introduce FODSeg, a voxel-based segmentation method that utilizes the complete fODF representation for each voxel, capturing the full angular structure of white matter orientation. Additionally, we reformulate tract segmentation as a singleclass problem, training one model per tract to reduce label conflicts inherent in multi-class approaches. This combination allows FODSeg to better distinguish tracts with similar local orientations and improves robustness in regions with structural ambiguity. We evaluate FODSeg on the Human Connectome Project dataset across all 72 white matter tracts using six segmentation accuracy metrics.

**Results:**

FODSeg achieves higher Dice scores and lower volumetric overreach values in 70% of the tracts while maintaining high specificity. Our results demonstrate the superior performance of FODSeg over existing segmentation approaches. Notably, our method shows significant improvements in anatomically challenging bottleneck regions, reducing false positives and improving tract-specific precision.

**Discussion:**

Overall, FODSeg advances white matter tract segmentation by leveraging the full richness of the fODF signal while improving accuracy, specificity, and anatomical consistency.

## 1 Introduction

White matter tract segmentation is a key step in diffusion MRI (dMRI) analysis, enabling the non-invasive reconstruction of major white matter pathways *in vivo* (Basser et al., 2000). These tracts are bundles of myelinated axons that function as communication pathways connecting different regions of the brain and supporting essential functions. Accurate segmentation of white matter tracts is essential for clinical applications, such as neurosurgical planning, as well as for research on brain development and neurological disorders (Bells et al., 2011; Chandio et al., 2020; Dayan et al., 2016). Among recent advances, deep learning-based direct voxel-based methods have emerged as an effective alternative to traditional tractography-based segmentation pipelines (Joshi et al., 2024). These approaches learn to assign a tract label to each voxel in the diffusion space, producing tract segmentations directly from the dMRI images, without requiring streamline generation or post-processing. Such methods have become integral for reliable and reproducible delineation of white matter tracts in the human brain (Kebiri et al., 2023; Wasserthal et al., 2018).

Despite significant advancements, white matter tract segmentation remains limited by fundamental challenges, including the well-documented crossing fiber problem (Alexander and Seunarine, 2010; Tournier, 2010) and the relatively underexplored bottleneck problem (Calixto et al., 2025; Schilling et al., 2022). The crossing-fiber problem typically refers to the case in which two or more differently oriented white matter tracts are located in the same imaging voxel; this causes a partial volume effects that lead to ambiguous or incorrect estimates of fiber orientations (Wheeler-Kingshott and Cercignani, 2009). This problem has been shown to occur in majority of the voxels in the brain (Jeurissen et al., 2013). While significant effort has gone into developing advanced diffusion models to estimate multiple orientations per voxel (Karimi et al., 2021; Seunarine and Alexander, 2014; Tournier et al., 2010), segmentation models still face difficulties resolving overlapping fibers in these complex regions. In the bottleneck problem, tracts enter the bottleneck regions along distinct orientations, merge and trace a specific distance in one or more voxels along the same orientation and then diverge towards distinct endpoints in the gray matter (Maier-Hein et al., 2017; Schilling et al., 2019). In such regions, segmentation models can struggle to correctly assign labels, often leading to misclassifications that can propagate errors across tract boundaries. Both problems introduce substantial ambiguity, increasing the risk of false positives and reducing the reliability of segmentations in anatomically complex zones.

In practice, segmentation models often use only the top three peaks of the fiber orientation distribution function (fODF) as input. The fODF describes the distribution of fiber orientations within a voxel and is typically represented as a function on a sphere, where peaks indicate dominant fiber directions. Using only a few dominant peaks, however, means that important orientation details may be lost in regions with complex fiber configurations, such as regions with highly crossing, kissing, or bending fibers. In contrast, using the full fODF retains a more complete picture of fiber distribution, which can be beneficial in distinguishing tracts that share similar orientations in certain voxels before diverging. By doing so, the model can learn to dynamically determine the most probable fiber orientation from the complete orientation distribution rather than being constrained to a predefined set of peaks.

Moreover, fODF-based multiclass segmentation methods may increase misclassifications. Since multiple tracts may share similar orientations within bottleneck regions as well as in crossing-fiber configurations, assigning a voxel to more than one tract can result in false positives and spatial inconsistencies. In this work, we aim to enhance voxel-based segmentation accuracy by employing strategies that mitigate these errors, ensuring more reliable tract identification, particularly in regions prone to crossing fibers and structural overlap. Unlike multi-class segmentation, where all tracts are predicted simultaneously, for a fixed backbone and realistic capacity, single-class segmentation focuses on one tract at a time, reducing inter-tract competition and label inconsistencies. This strategy can mitigate false positives, particularly in regions where multiple tracts intersect, because the model is not forced to assign a voxel to multiple tracts in a single forward pass. Additionally, single-class segmentation can better capture tract-specific features and adapt to variations in tract anatomy across subjects.

We hypothesize that the full directional information provided by fODFs, combined with a single-class segmentation approach, will improve tract-specific fidelity and enhance the overall reliability of tract segmentations. By systematically evaluating our approach on a public dataset of major white matter tracts, we assess its effectiveness in mitigating bottleneck-related artifacts and crossing fibers. Our goal is to effectively decrease false-positive results and improve overall segmentation accuracy, ensuring more reliable and anatomically faithful tract segmentations, which may ultimately enhance the interpretability of downstream structural connectivity and clinical analyses. Our contributions can be summarized as follows:

1. Full fODF utilization: Instead of relying on a limited number of dominant peaks per voxel, as done in existing methods, we propose using the entire fODF obtained through constrained spherical deconvolution.
2. Single-class segmentation for improved accuracy: We reformulated the white matter tract segmentation task as a single-class problem rather than a multi-class problem.
3. Comprehensive evaluation beyond dice scores: We conduct an extensive evaluation to assess the efficiency and effectiveness of our proposed model. In addition to Dice scores, we incorporate volumetric overlap and overreach metrics to provide a more nuanced understanding of segmentation performance and provide qualitative evaluations.

## 2 Related work

Direct voxel-based segmentation of white matter bundles predates deep learning, with early work proposing level-set formulations (Feddern et al., 2006), graph or Markov random field (MRF) models (Awate et al., 2007; Wang and Vemuri, 2005), and orientation density function (ODF)-based tract segmentation frameworks (Hagmann et al., 2006). Building on these foundations, deep learning–based direct segmentation approaches simplify the white matter tract segmentation pipeline by directly producing complete tract segmentations from input dMRI images. Several automated direct segmentation methods have been developed in recent years (Joshi et al., 2024), with TractSeg (Wasserthal et al., 2018; Wasserthal et al., 2019) being the most widely adopted tool. TractSeg relies on fODF peaks as input derived from constrained spherical deconvolution (CSD), using three dominant peaks per voxel to estimate tract segmentations. While fODF peaks-based methods offer structured fiber orientation modeling, they inherently discard weaker but potentially meaningful fiber directions. Because CSD encodes the same local orientation ambiguities that underlie tractography difficulties, voxel-wise models are also exposed to crossing-fiber and bottleneck-related uncertainty. Some approaches (Kebiri et al., 2023; Lucena et al., 2022) attempt to overcome the limitations of peak-based methods by using spherical harmonics (SH) coefficients directly, without explicitly estimating fODFs. While both SH-based signal representations and fODF-based techniques utilize spherical harmonics, fODF methods explicitly model fiber orientation distributions through deconvolution, enhancing their interpretability and effectiveness in resolving crossing fibers compared to methods that rely solely on SH signal representation (Tournier et al., 2007). This trade-off between preserving raw signal information and enforcing orientation structure remains an open challenge in tract segmentation.

To improve segmentation accuracy, multimodal approaches have been explored by integrating diffusion data with structural MRI modalities. Some methods incorporate T1-weighted images alongside fODF peaks (Dong et al., 2019), while others combine T1-weighted images with principal diffusion directions (Nelkenbaum et al., 2020) or fractional anisotropy (FA) maps (Li et al., 2021) as additional inputs. These strategies aim to provide complementary anatomical information to enhance segmentation. However, they often fail to generalize when segmenting all 72 white matter tracts, either showing minimal performance improvements or being tested only on a subset of tracts. Alternative approaches have been proposed that avoid fODFs entirely. Some methods use entire 4D diffusion tensor images (DTI) as input (Li et al., 2020), while others rely on whole-brain diffusion anisotropy maps (Pomiecko et al., 2019). These models typically train a separate network for each tract; however, despite their distinct formulations, their segmentation accuracy remains comparable to TractSeg and does not extend to all 72 tracts, limiting their applicability for comprehensive whole-brain tractography. Among deep-learning-based segmentation methods, the 2D U-Net architecture has become the most widely adopted framework, often with minor modifications to the baseline U-Net design (Yin et al., 2022). U-Net-based models achieve strong performance in many cases, effectively capturing local spatial features while maintaining computational efficiency, making it a popular choice for direct segmentation approaches.

In summary, most direct segmentation methods employ fODF peaks or multimodal fusion strategies to enhance tract segmentation. While these approaches yield satisfactory performance, they remain limited in their ability to address crossing fiber interactions and bottleneck regions. Currently, few deep-learning-based segmentation models explicitly tackle these challenges, highlighting the need for novel approaches that improve robustness in whole-brain tract segmentation.

## 3 Methodology

**Figure 1:**
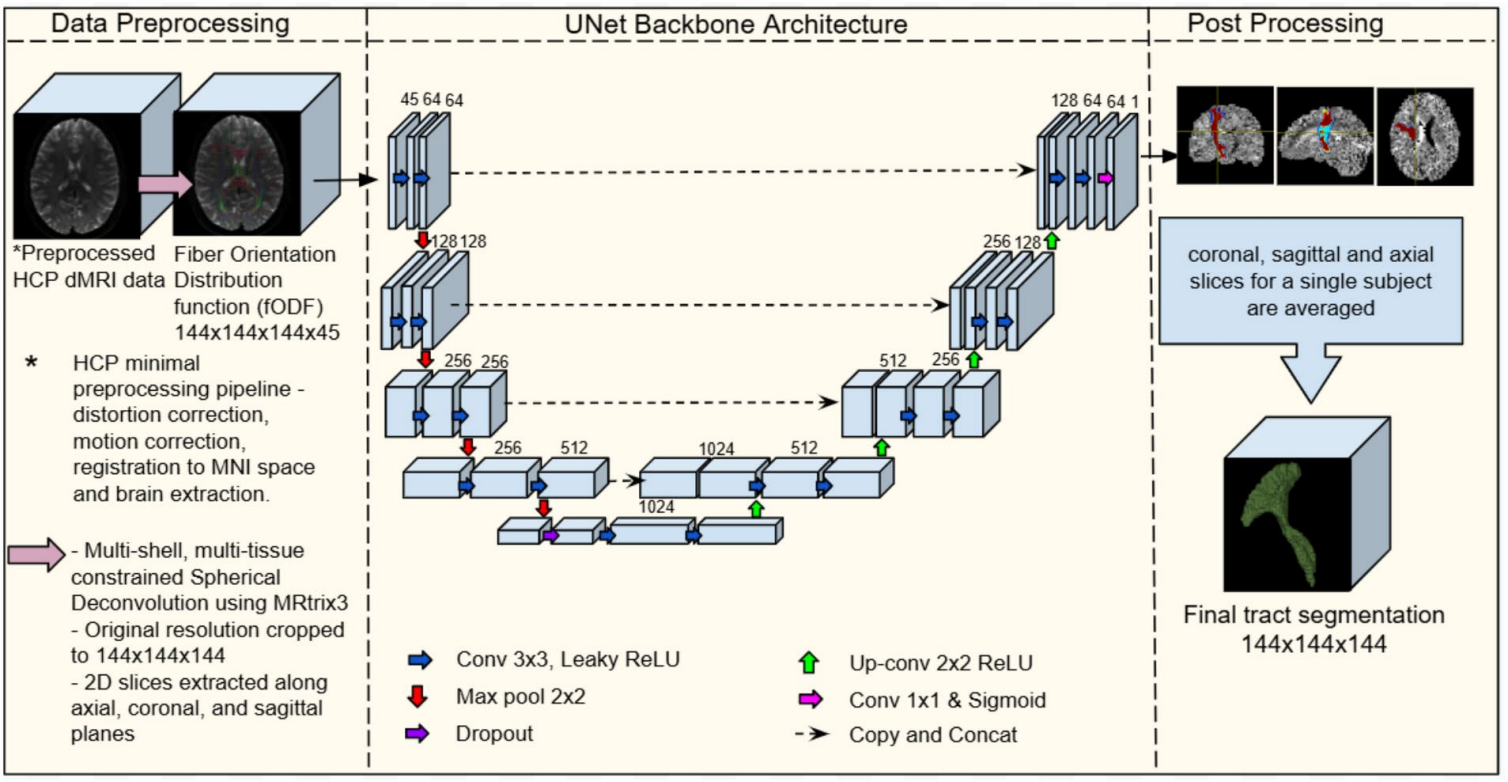
Overview of the FODSeg single class segmentation framework. The input consists of preprocessed diffusion MRI (dMRI) scans from the HCP dataset, which undergo minimal preprocessing. Fiber orientation distribution functions (fODFs) are computed using multi-shell, multi-tissue constrained spherical deconvolution (MSMT-CSD) via MRtrix3 and cropped to a resolution of 144 × 144 × 144 × 45. A single 2D slice of 144 × 144 × 45 from the fODF volume is fed into a 2D U-Net. The network outputs a binary segmentation mask for the tract of interest. During inference, segmentations are generated independently along the axial, sagittal, and coronal views. The three resulting probability maps are averaged voxel-wise and thresholded at 0.5 to obtain the final 3D tract segmentation. The tract shown is the right corticospinal tract from a representative subject. Illustrations of the right corticospinal tracts and brain shape come from a single subject.

Figure 1 presents an overview of our proposed FODSeg framework. The details specific to the illustrated framework are given below.

### 3.1 Data preprocessing

We began our training and evaluation using dMRI scans from the Human Connectome Project (HCP) dataset (Van Essen et al., 2013; Sotiropoulos et al., 2013). These scans were acquired with 270 diffusion gradient directions across three *b*-values (1,000, 2,000, and 3,000 s/mm^2^), with an isotropic spatial resolution of 1.25 mm. All scans were preprocessed using HCP’s minimal preprocessing pipeline (Glasser et al., 2013). In total, dMRI data from 105 subjects were used in this study.

For each subject, fODFs were computed voxel-wise by fitting a constrained spherical deconvolution (CSD) model (Tournier et al., 2007) using MRtrix3 (Tournier et al., 2019). The fODFs are transformed into the spherical harmonic (SH) domain, which constitutes a natural representation for spherical functions, with a maximum order of 8, resulting in 45 real SH coefficients per voxel (Tournier et al., 2004). To ensure robustness across different CSD configurations, fODFs were extracted using both (1) multi-shell, multi-tissue CSD with all gradient directions and (2) standard single-shell CSD using only *b* = 1,000 s/mm^2^ gradient directions. All FODSeg models were trained under these conditions. To standardize input dimensions across the dataset, the original HCP images with a resolution of 145 × 174 × 145 were centrally cropped to a uniform shape of 144 × 144 × 144 (Wasserthal et al., 2018). The final HCP images therefore have a size of 144 × 144 × 144 with 45 channels. Each subject also had 3D binary masks for 72 white matter tracts, manually corrected and provided as part of the dataset (Wasserthal et al., 2018) and are available for download.^1^

We used the 105 HCP subjects and 72 expert-corrected tract reference masks released with TractSeg as our only dataset and as ground truth. Following the official recommendations provided with the TractSeg dataset these subjects are partitioned into five folds (fold1–fold5, each with 21 subjects). In all experiments, we train on fold1 + fold2 + fold3 (63 subjects), select hyperparameters using fold4 (21 subjects), and report test results on fold5 (21 subjects). No subject appears in more than one split.

The 3D fODF volumes were converted into 2D slices for network input by extracting views along the axial, coronal, and sagittal planes. Each 2D slice had a spatial resolution of 144 × 144 and retained the 45 SH channels. The segmentation outputs were generated in the same shape and format for each view.

### 3.2 Backbone network architecture

The proposed 2D encoder-decoder convolutional neural network is based on the widely adopted U-Net architecture, which has proven effective in various biomedical segmentation tasks. Although our method is architecture-agnostic and can be adapted to alternative segmentation backbones, we utilize a standard U-Net structure in this work for consistency and reproducibility.

The encoder part of the U-Net uses 2D convolutions with a kernel size of 3 × 3 and a stride of 1, followed by the Leaky ReLU activation functions and MaxPool with kernel size 2 × 2 for downsampling. The number of filters used in each layer is shown in Figure 1. The decoder mirrors the encoder with the convolution replaced by the transposed convolution for upsampling, along with a skip connection to the corresponding encoder block at the same resolution level. The network is trained using binary cross-entropy loss.

### 3.3 Implementation details

*Training strategy*: Each tract was trained as an independent binary segmentation task. Voxels belonging to multiple tracts in the ground truth were retained in the positive class for all relevant tracts, allowing overlapping regions to be represented in multiple single-tract models. This design reduces inter-tract competition present in multi-class setups and enables each model to learn tract-specific features even in overlapping or bottleneck regions.

The proposed method is implemented in PyTorch (Paszke et al., 2019). All models are trained on an Ubuntu system equipped with an NVIDIA RTX 4090 GPU with 24 GB memory. We use the Adam optimizer with a batch size of 200 and train for up to 400 epochs with dropout set to 0.4. MONAI 1.3.0 is used for data loading and sampling. For model development and evaluation, we adopted a validation strategy with subject-wise splits, allocating 63 subjects for training, 21 for validation, and 21 for testing. The full training code is publicly available as an open-source project.^2^

*Hyperparameter optimization*: To ensure robust training and generalization, we conducted systematic exploration of key hyperparameters, guided by prior work and exploratory validation experiments. We varied the learning rate in the range of 1 × 10^−3^ to 1 × 10^−5^ and adopted a cosine annealing schedule, with the final choice of an initial learning rate of 0.002 providing the best trade-off between convergence speed and stability. Batch size was tested between 100 and 400, with 200 yielding stable gradient updates while fitting within GPU memory constraints. We also assessed training length up to 500 epochs, using early stopping based on validation Dice with a patience of 50 epochs. The model checkpoint with the highest Dice score on the validation set is used for testing.

### 3.4 Dataset postprocessing

Following segmentation, postprocessing steps were applied to ensure consistency and quality across the generated tract masks. First, for each subject, the 2D segmentation outputs from the axial, sagittal, and coronal planes were combined by averaging the predicted probabilities at each voxel across all three views. A final 3D binary mask was then obtained by thresholding the averaged output at 0.5.

### 3.5 Evaluation metrics

We evaluate segmentation performance using a comprehensive set of voxel-level metrics that together capture different aspects of accuracy and error. These include Dice score, volumetric overlap (VOP), volumetric overreach (VOR), specificity, precision, and the Jaccard index. While most of these metrics are widely used, VOP and VOR are particularly important. VOP measures the fraction of the reference tract volume correctly recovered by the segmentation and VOR directly quantifies the proportion of false positives in the predicted segmentation (Maier-Hein et al., 2017). Lower VOR values indicate greater spatial specificity, making it especially useful for assessing segmentation reliability in anatomically complex or overlapping regions. VOP and VOR are therefore complementary; high VOP with low VOR indicates both good coverage and high specificity. The description, formulation and usage for each of the metrics is summarized in the Supplementary Table S1.

### 3.6 Baseline

We benchmark our method with TractSeg (Wasserthal et al., 2018), based on the TractSeg codebase^3^ (version 2.9), a widely adopted and high-performing direct tract segmentation approach. Prior work (Joshi et al., 2024) has summarized multiple direct segmentation approaches and shows that among methods evaluated on all 72 tracts, TractSeg consistently demonstrates the strongest reported performance, as measured by Dice. Therefore, we adopt TractSeg as the principal baseline for comparison in this work.

For a fair comparison, we applied the preprocessing pipeline recommended by TractSeg on the HCP dataset and used the latest version of the pretrained weights released by TractSeg to generate tract predictions. Final evaluation scores for TractSeg were computed using the same test dataset and evaluation metrics as those used for our proposed method. We also adopted the same dataset partitioning, data augmentation, and experimental settings as used in TractSeg to ensure a consistent comparison. The intensity of data augmentations was varied randomly, and were applied online during training, consistent with the default TractSeg settings.

## 4 Experiments and results

### 4.1 Full fODF utilization & single-class segmentation

To evaluate the effectiveness of our proposed approach, we designed a two-part experiment comparing our approach against the TractSeg method (Wasserthal et al., 2018). TractSeg performs multi-class segmentation using three fODF peaks as input features. In the first part of the experiment, we replicate the multi-class segmentation setup using our proposed full fODF representation as input, enabling us to assess the advantage of utilizing the complete orientation information over peak-based representations. This model is denoted as FODSeg_mc. In the second part of the experiment, we evaluate the benefits of shifting from multi-class to single-class segmentation. We train independent binary segmentation models for each white matter tract using the same full fODF input representation. This configuration, referred to as FODSeg_sc, allows the model to focus better on individual tract structures and reduces inter-class competition, which can be particularly beneficial in anatomically complex regions with overlapping tracts. We evaluate all methods on the same test set using a consistent pipeline and report the average performance across all tracts using six segmentation metrics. The Wilcoxon signed-rank test was used to test statistical significance with Bonferroni correction for multiple testing.

The results are summarized in Table 1. Compared to the baseline TractSeg, our multi-class model (FODSeg_mc) shows modest improvements in Dice score, precision, and Jaccard index, indicating better overall segmentation accuracy and tract localization. When transitioning to single-class segmentation (FODSeg_sc), we observe the highest Dice and Jaccard scores among all methods, along with consistently high specificity. Notably, the single-class model achieves this while maintaining a low volumetric overreach (VOR), suggesting improved control over false positives.

**Table 1:** Comparison of average segmentation performance across all 72 white matter tracts on the 21-subject fold5 test set from the TractSeg HCP dataset for three methods: TractSeg, FODSeg_multi-class (FODSeg_mc), and FODSeg_single-class (FODSeg_sc).

Method	Dice↑	VOP↑	VOR↓	Specificity↑	Precision↑	Jaccard↑
TractSeg	0.829 ± 0.06	0.854 ± 0.06	0.211 ± 0.19	0.998 ± 0.00	0.813 ± 0.08	0.712 ± 0.07
FODSeg_mc	0.835 ± 0.06	0.820 ± 0.07	**0.146 ± 0.16**	**0.999 ± 0.00**	**0.859 ± 0.08**	0.721 ± 0.07
FODSeg_sc	**0.844 ± 0.05** ^†^	**0.859 ± 0.04**	0.18 ± 0.17	**0.999 ± 0.00**	0.833 ± 0.07	**0.737 ± 0.07**

† **Wilcoxon** signed-rank tests on Dice scores across all 72 tracts show: FODSeg_mc > TractSeg (*p* = 1.94e^−7^), FODSeg_sc > TractSeg (*p* = 2.51e^−34^), and FODSeg_sc > FODSeg_mc (*p* = 1.55e10^−98^).

Results are reported as mean ± standard deviation across all subjects for six evaluation metrics. Arrows (↑ / ↓) indicate whether higher or lower values are better for each metric. Values in bold represent the top-performing method based on metric.

**Figure 2:**
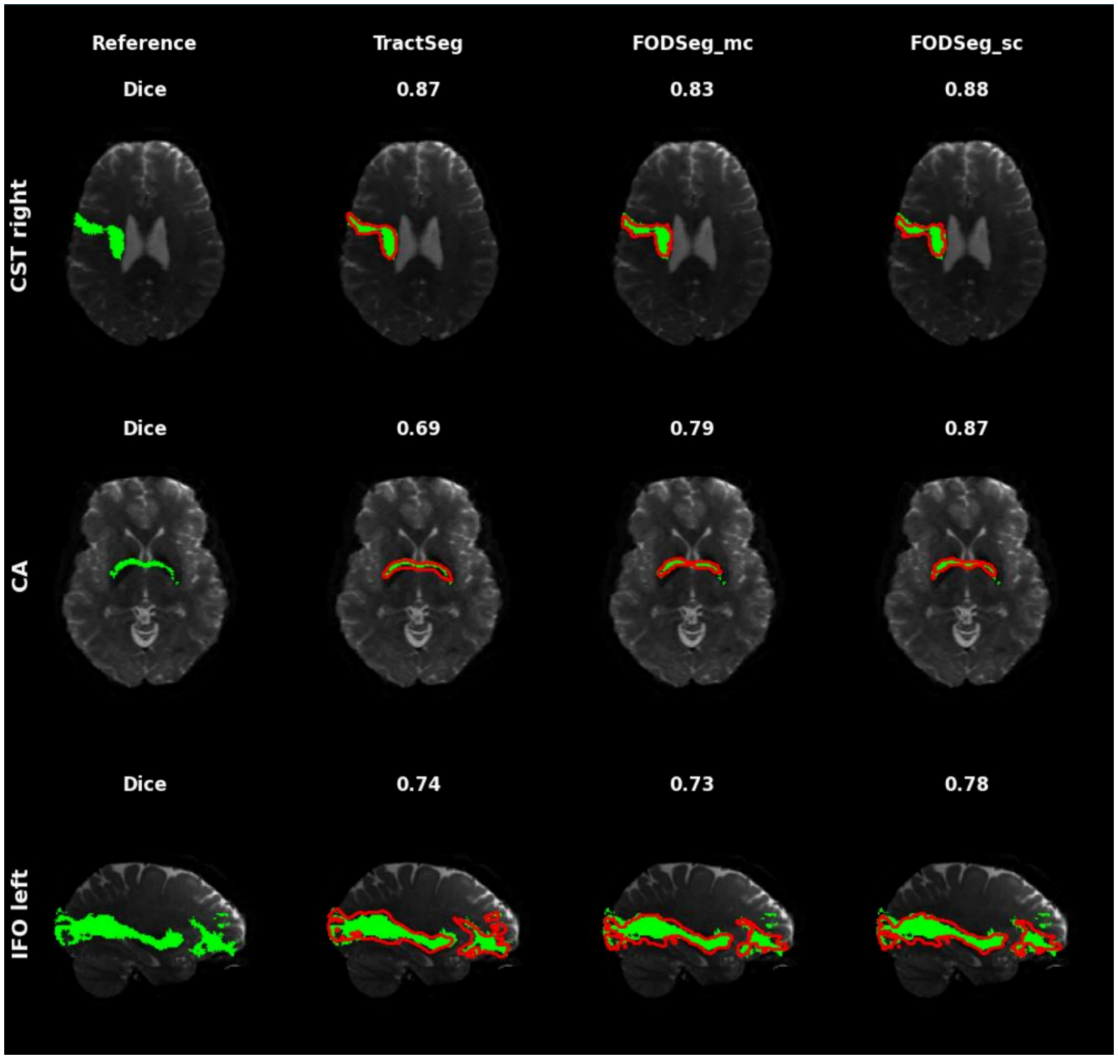
Qualitative comparison of tract segmentation results from a randomly selected subject in the HCP test dataset. The first column shows the reference (ground truth) tracts, which were generated from the HCP dataset using a semi-automated method and manually corrected by experts, as provided in the TractSeg release (Wasserthal et al., 2018). The second, third, and fourth columns display the segmentation outputs from TractSeg, FODSeg_mc, and FODSeg_sc, respectively. The green regions indicate the reference tracts, while the red regions represent segmentations from each method. The Dice scores displayed correspond to the shown 2D axial or sagittal slice.

To qualitatively assess segmentation performance across tracts with varying levels of reconstruction difficulty, we followed the evaluation strategy proposed by TractSeg and visualized results for three representative tracts, the inferior fronto-occipital fasciculus (IFO), corticospinal tract (CST), and commissure anterior (CA). These tracts span a spectrum of difficulty levels, IFO being relatively easy to reconstruct with consistently high accuracy across methods, CST presenting moderate difficulty due to its fanning cortical projections, and CA being highly challenging due to its thin, elongated structure connecting the temporal lobes. Figure 2 displays qualitative segmentations for these tracts across TractSeg, FODSeg_mc, and FODSeg_sc, allowing for visual comparison of performance in both well-defined and anatomically complex cases. As can be seen, our proposed method FODSeg_sc yielded adequate reconstructions on all the tracts.

### 4.2 Tract-wise evaluation

**Figure 3:**
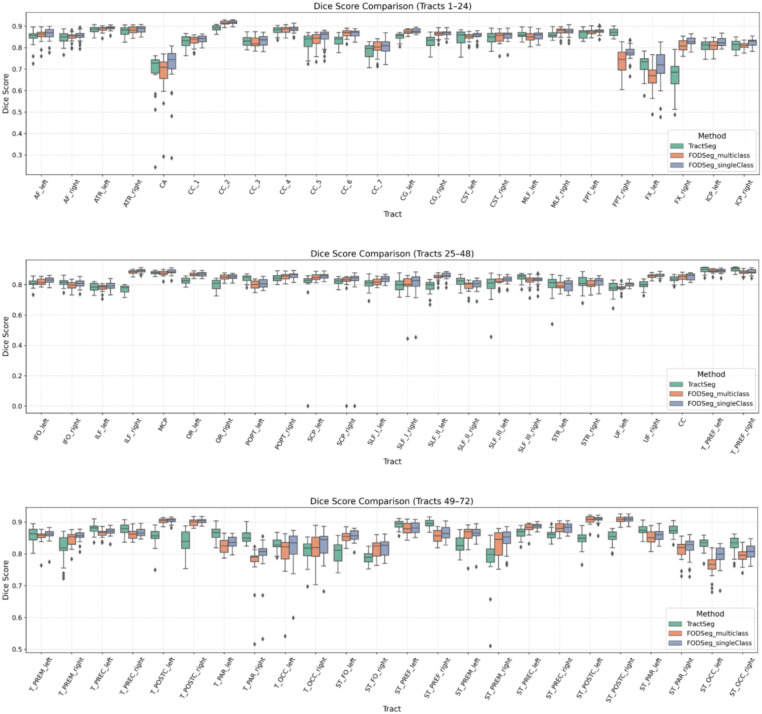
Dice scores for all 72 white matter tracts across 21 target subjects for TractSeg, FODSeg_mc, and FODSeg_sc.

To evaluate the segmentation performance across individual white matter tracts, we first conducted a tract-wise comparison between TractSeg, FODSeg_mc, and our proposed FODSeg_sc model. Boxplots showing the Dice scores for all 72 tracts across TractSeg, FODSeg_mc, and FODSeg_sc are presented in Figure 3. Each boxplot represents the distribution of Dice scores across subjects for a given tract, enabling detailed comparison of segmentation performance at the individual tract level. This analysis quantifies the segmentation quality for each of the 72 tracts in the HCP dataset and allows for a detailed comparison of performance across different methods.

In addition to the full set, and motivated by prior benchmarking studies (Berto et al., 2021; Lucena et al., 2022), we present more detailed quantitative results for a subset of clinically relevant tracts commonly evaluated in the literature (Lucena et al., 2022). Specifically, we report Dice scores for five major tracts in both the left and right hemispheres: arcuate fasciculus (AF), CST, IFO, inferior longitudinal fasciculus (ILF), and uncinate fasciculus (UF). These tracts are part of the standard evaluation protocol used in several recent studies and their results can be directly compared with previously published state-of-the-art methods. Specifically, RecoBundles is a streamline-based method that performs segmentation via clustering and streamline matching against reference bundles (Garyfallidis et al., 2015). U-Net refers to the 3D U-Net implementation (Çiçek et al., 2016) provided within the nnU-Net framework (Isensee et al., 2019). And finally, Classifyber is a streamline-based method that uses binary classifiers to assign streamlines from a whole-brain tractogram to individual bundles (Berto et al., 2021). The tract-wise Dice performance for our method and other approaches is summarized in Table 2.

**Table 2:** Comparison with state-of-the-art approaches for the following 5 tracts from both left and right sides of the brain using Dice: arcuate fascicle (AF), corticospinal tract (CST), inferior fronto-occipital fascicle (IFO), inferior longitudinal fascicle (ILF), and uncinate fascicle (UF).

Tract	RecoBundles^a^	TractSeg^b^	U-Net^c^	FODSeg_mc	Classifyber^d^	FODSeg_sc
Right CST	0.62 ± 0.07	0.85 ± 0.02	0.84 ± 0.03	0.85 ± 0.01	**0.87** ± **0.02**	0.85 ± 0.02
Left CST	0.62 ± 0.11	0.85 ± 0.03	0.85 ± 0.03	0.85 ± 0.01	**0.86** ± **0.10**	0.85 ± 0.02
Right UF	0.57 ± 0.24	0.79 ± 0.03	0.77 ± 0.04	0.85 ± 0.01	**0.86** ± **0.03**	**0.86 ± 0.01**
Left UF	0.55 ± 0.27	0.77 ± 0.03	0.75 ± 0.07	0.78 ± 0.02	**0.84** ± **0.04**	0.80 ± 0.01
Right AF	0.53 ± 0.11	0.83 ± 0.02	0.83 ± 0.03	0.85 ± 0.03	**0.86** ± **0.03**	**0.86 ± 0.02**
Left AF	0.71 ± 0.05	0.84 ± 0.03	0.84 ± 0.03	0.85 ± 0.02	0.83 ± 0.04	**0.86 ± 0.03**
Right ILF	0.42 ± 0.13	0.75 ± 0.03	0.80 ± 0.03	0.88 ± 0.01	0.82 ± 0.04	**0.89 ± 0.01**
Left ILF	0.57 ± 0.07	0.77 ± 0.02	0.80 ± 0.03	0.78 ± 0.02	**0.84** ± **0.04**	0.79 ± 0.02
Right IFO	0.76 ± 0.04	0.80 ± 0.02	0.80 ± 0.04	0.79 ± 0.02	**0.84** ± **0.03**	0.80 ± 0.02
Left IFO	0.67 ± 0.06	0.80 ± 0.02	0.78 ± 0.04	0.82 ± 0.02	**0.84** ± **0.03**	0.83 ± 0.01

a Garyfallidis et al. (2015).

b Wasserthal et al. (2018).

c Lucena et al. (2022).

d Berto et al. (2021).

Results for TractSeg, ReconBundles and Classifyber are reported in (Berto et al., 2021). All methods are evaluated on the 21-subject fold5 test set of the TractSeg dataset. Values in bold represent the top-performing method based on Dice score. Results are reported as mean ± standard deviation.

### 4.3 Bottleneck regions evaluation

To assess the effectiveness of our method in anatomically complex regions, we evaluated segmentation performance in specific white matter bottleneck areas, regions where different tracts share a similar location and orientation yet have distinct origins and terminations, making accurate tract segmentation particularly challenging. Bottleneck areas have recently been quantified (Calixto et al., 2025; Schilling et al., 2022), with evidence showing that they occur in over 50–70% of fixels in the white matter of the human brain. In this work, we focus on specific tracts in three such bottleneck regions. The first bottleneck region, characterized by the highest density of converging tracts, is located in the deep white matter of the occipital lobe with anterior–posterior orientation. It includes tracts such as the splenium of the corpus callosum (CC_7), optic radiation (OR), and the middle longitudinal fasciculus (MLF), among others. The second major bottleneck region comprises tracts oriented in the superior–inferior direction within the brainstem, including T_PREM (thalamo-premotor), STR (superior thalamic radiations), ST_POSTC (striato-postcentral), and POPT (parieto-occipital pontine). A third bottleneck zone, located in the superior–inferior oriented white matter of the internal capsule, includes tracts like the CST and POPT.

Segmentation quality is assessed using the same evaluation metrics and setup described previously. A quantitative summary of the performance across these tracts is presented in Table 3. For brevity, evaluation metrics including specificity, precision, and Jaccard index are provided in the Supplementary Table S2. As seen, FODSeg achieves higher Dice scores for 8 out of 10 tracts. VOP is also higher or comparable for most tracts. VOR is consistently lower or equal in 8 out of 10 tracts with FODSeg, indicating fewer false positives. Comparable results are observed in STR and CST, where Dice and VOR values remain unchanged across both methods.

**Table 3:** Quantitative comparison of segmentation performance for selected tracts passing through bottleneck regions.

Bottleneck region	Tract	Dice↑			VOR↓		
		TractSeg	FODSeg_mc	FODSeg_sc	TractSeg	FODSeg_mc	FODSeg_sc
Occipital (anterior–posterior)	CC_7	0.78 ± 0.03	0.79 ± 0.03	**0.80 ± 0.03**	0.31 ± 0.10	**0.22 ± 0.11**	0.25 ± 0.08
	OR	0.81 ± 0.02	0.85 ± 0.01	**0.86 ± 0.01**	0.24 ± 0.07	**0.13 ± 0.05**	0.15 ± 0.02
	MLF	**0.86 ± 0.01**	0.86 ± 0.02	**0.86 ± 0.02**	0.17 ± 0.04	**0.14 ± 0.06**	0.15 ± 0.04
	IFO	0.80 ± 0.02	0.80 ± 0.02	**0.82 ± 0.01**	0.23 ± 0.08	**0.17 ± 0.09**	0.20 ± 0.04
Brainstem (superior–inferior)	T_PREM	0.83 ± 0.02	0.85 ± 0.02	**0.86 ± 0.02**	0.17 ± 0.03	**0.10 ± 0.04**	0.14 ± 0.04
	STR	**0.80 ± 0.06**	0.79 ± 0.02	**0.80 ± 0.03**	0.25 ± 0.25	**0.19 ± 0.11**	0.25 ± 0.11
	ST_POSTC	0.85 ± 0.02	0.90 ± 0.01	**0.91 ± 0.01**	0.16 ± 0.04	**0.08 ± 0.03**	0.09 ± 0.03
Internal capsule (superior–inferior)	CST	**0.85 ± 0.03**	0.84 ± 0.02	**0.85 ± 0.02**	0.14 ± 0.04	**0.12 ± 0.05**	0.14 ± 0.05
	ST_PREF	**0.89 ± 0.01**	0.86 ± 0.02	0.87 ± 0.01	0.12 ± 0.03	**0.12 ± 0.06**	0.15 ± 0.04
	POPT	**0.84 ± 0.02**	0.82 ± 0.03	0.83 ± 0.02	0.21 ± 0.07	**0.16 ± 0.09**	0.19 ± 0.07

The results are shown for TractSeg, FODSeg_mc (multiclass) and the FODSeg_sc (single class) segmentation methods across two evaluation metrics. The results for the other metrics are provided in the Supplementary Table S2. Tracts were selected due to their anatomical complexity and prevalence in major bottleneck regions of the white matter, where accurate segmentation is particularly challenging. The best-performing scores for each metric are highlighted in bold.

### 4.4 Clinical quality data evaluation

#### 4.4.1 Single-shell fODF analysis

To assess the robustness of FODSeg to different fODF estimation strategies and to better approximate clinical-quality data, we conducted experiments using fODFs derived from standard single-shell CSD using only *b* = 1,000s/mm^2^. For FODSeg_sc, we generated full single-shell fODFs (standard CSD, *b* = 1,000 s/mm^2^ only) and retrained the network from scratch on these inputs using the 63 training subjects, with validation on 21 subjects. Both TractSeg and FODSeg models were tested on the same single-shell held-out 21-subject fold. The same dataset partitioning, preprocessing pipeline, and training protocol as in the main experiments were employed to ensure comparability. We benchmarked our results against TractSeg. For TractSeg, we computed MRtrix CSD peaks from the same single-shell *b* = 1,000 s/mm^2^ data and evaluated the official pretrained TractSeg model (pretrained_weights_tract_segmentation_v3.npz) without any additional fine-tuning. This pretrained model had originally been trained on the 105 HCP subjects using three peak configurations, multi-shell multi-tissue shell data, standard *b* = 1,000 s/mm^2^, and 12-direction *b* = 1,000 s/mm^2^ shell. The results are summarized in Table 4. Results show that FODSeg_sc achieves performance that is better or comparable to TractSeg across both tract-wise and bottleneck evaluations, despite the reduced information available from single-shell acquisitions. Remarkably, even when trained only on single-shell data, FODSeg_sc achieves comparable performance, highlighting the robustness of our framework under more constrained acquisition settings.

**Table 4:** Comparison of TractSeg and FODSeg_sc trained using single-shell (*b* = 1,000 s/mm^2^) CSD data.

Tracts		Dice↑	
		TractSeg	FODSeg_sc
Right CST		**0.85 ± 0.02**	**0.85 ± 0.02**
Left CST		**0.85 ± 0.03**	0.84 ± 0.03
Right UF		**0.79 ± 0.03**	**0.79 ± 0.02**
Left UF		**0.77 ± 0.03**	**0.77 ± 0.03**
Right AF		0.83 ± 0.02	**0.85 ± 0.02**
Left AF		0.84 ± 0.03	**0.86 ± 0.03**
Right ILF		0.75 ± 0.03	**0.77 ± 0.02**
Left ILF		0.77 ± 0.02	**0.79 ± 0.02**
Right IFO		0.80 ± 0.02	**0.81 ± 0.02**
Left IFO		0.80 ± 0.02	**0.81 ± 0.02**
Occipital (anterior–posterior)	CC_7	**0.79 ± 0.03**	0.78 ± 0.02
	OR	**0.85 ± 0.01**	0.82 ± 0.02
	MLF	0.86 ± 0.02	**0.87 ± 0.01**
	IFO	0.80 ± 0.02	**0.81 ± 0.02**
Brainstem (superior–inferior)	T_PREM	**0.85 ± 0.02**	0.83 ± 0.02
	STR	0.79 ± 0.02	**0.80 ± 0.05**
	ST_POSTC	**0.90 ± 0.01**	0.85 ± 0.02
Internal capsule (superior–inferior)	CST	0.84 ± 0.02	**0.85 ± 0.02**
	ST_PREF	0.86 ± 0.02	**0.90 ± 0.02**
	POPT	0.82 ± 0.03	**0.85 ± 0.02**

Dice scores (mean ± SD) are reported for clinically relevant tracts (top), and tracts passing through major bottleneck regions (bottom). The best-performing scores are highlighted in bold.

#### 4.4.2 Downsampled clinical quality analysis

To further evaluate the robustness of our framework under conditions that approximate clinical diffusion MRI acquisitions, we generated a Clinical-Quality dataset by downsampling the HCP data to an isotropic resolution of 2.5 mm and retaining only 32 gradient directions at *b* = 1,000 s/mm^2^. This setting mimics the typical clinical protocols that use lower angular resolution and fewer diffusion volumes than research-grade acquisitions. Reference tracts from the original HCP dataset were reused as ground truth to enable proper evaluation on the downsampled data. Since this dataset contains only a single *b*-value shell, standard CSD was applied for computing fODFs. We then fine-tuned our existing FODSeg models on full fODFs derived from this downsampled dataset (63 training subjects, 21 validation subjects) and evaluated on the same 21-subject test split for 100 epochs using a ReduceLROnPlateau scheduler. Wasserthal et al. (2018) also trained a separate TractSeg model specifically on downsampled “clinical-quality” data; however, those clinical-specific weights are not publicly available. In our experiments, TractSeg is therefore evaluated using the same official HCP-trained model as above, applied to MRtrix peaks computed from the 2.5 mm, 32-direction data, without re-training. This configuration reflects TractSeg’s recommended pretrained usage but cannot reproduce the exact clinical-specific training reported in the original paper. Accordingly, we interpret this experiment primarily as a demonstration of FODSeg_sc’s adaptability to clinical-like acquisitions, while using TractSeg in its standard pretrained form for reference. For this experiment, we show the performance of TractSeg and FODSeg_sc models across all evaluation metrics. Results are summarized in Table 5. We also focus on the previously defined subset of representative and clinically relevant tracts, as well as tracts traversing bottleneck regions, to specifically assess performance in challenging anatomical locations. These results are summarized in Table 6. Compared to TractSeg, FODSeg_sc achieved higher Dice scores in all of the evaluated tracts, despite the lower resolution and reduced angular information.

**Table 5:** Comparison of average segmentation performance across all 72 white matter tracts in the HCP clinical quality dataset for the two methods: TractSeg, and FODSeg_sc (single class).

Method	Dice↑	VOP↑	VOR↓	Specificity↑	Precision↑	Jaccard↑
TractSeg	0.759 ± 0.06	0.782 ± 0.08	0.283 ± 0.19	0.998 ± 0.00	0.745 ± 0.08	0.616 ± 0.08
FODSeg_sc	**0.836 ± 0.05**	**0.844 ± 0.05**	**0.179 ± 0.16**	**0.999 ± 0.00**	**0.833 ± 0.07**	**0.722 ± 0.07**

Results are reported as mean ± standard deviation across all subjects for six evaluation metrics. Arrows (↑ / ↓) indicate whether higher or lower values are better for each metric. The best-performing scores for each metric are highlighted in bold.

**Table 6:** Dice scores (mean ± SD) and Volumetric Overreach (VOR) (mean ± SD) for TractSeg and FODSeg_sc (single class) on a subset of representative, clinically relevant (top), and bottleneck tracts (bottom) using the downsampled clinical-quality dataset (2.5 mm isotropic resolution, 32 gradient directions at *b* = 1,000 s/mm^2^).

Tracts		Dice↑		VOR↓	
		TractSeg	FODSeg_sc	TractSeg	FODSeg_sc
Right CST		0.78 ± 0.03	**0.84 ± 0.01**	0.18 ± 0.04	**0.16 ± 0.05**
Left CST		0.80 ± 0.03	**0.84 ± 0.03**	0.16 ± 0.03	**0.14 ± 0.05**
Right UF		0.73 ± 0.03	**0.84 ± 0.01**	0.27 ± 0.13	**0.14 ± 0.04**
Left UF		0.71 ± 0.04	**0.78 ± 0.01**	0.25 ± 0.14	**0.23 ± 0.06**
Right AF		0.77 ± 0.02	**0.84 ± 0.01**	0.26 ± 0.08	**0.15 ± 0.05**
Left AF		0.77 ± 0.03	**0.84 ± 0.02**	0.28 ± 0.09	**0.15 ± 0.05**
Right ILF		0.69 ± 0.02	**0.86 ± 0.01**	0.38 ± 0.12	**0.09 ± 0.02**
Left ILF		0.69 ± 0.02	**0.76 ± 0.02**	0.35 ± 0.13	**0.23 ± 0.06**
Right IFO		0.75 ± 0.02	**0.78 ± 0.02**	0.32 ± 0.08	**0.22 ± 0.08**
Left IFO		0.75 ± 0.02	**0.79 ± 0.02**	0.31 ± 0.10	**0.19 ± 0.05**
Occipital (anterior–posterior)	CC_7	0.71 ± 0.03	**0.77 ± 0.02**	0.50 ± 0.14	**0.26 ± 0.08**
	OR	0.74 ± 0.02	**0.82 ± 0.01**	0.35 ± 0.11	**0.15 ± 0.05**
	MLF	0.77 ± 0.01	**0.85 ± 0.01**	0.25 ± 0.05	**0.14 ± 0.03**
	IFO	0.75 ± 0.02	**0.79 ± 0.02**	0.32 ± 0.09	**0.21 ± 0.07**
Brainstem (superior–inferior)	T_PREM	0.78 ± 0.03	**0.84 ± 0.02**	0.21 ± 0.10	**0.15 ± 0.04**
	STR	**0.74 ± 0.06**	0.71 ± 0.03	0.28 ± 0.20	**0.17 ± 0.08**
	ST_POSTC	0.78 ± 0.02	**0.90 ± 0.01**	0.23 ± 0.05	**0.10 ± 0.02**
Internal capsule (superior–inferior)	CST	0.79 ± 0.03	**0.84 ± 0.02**	0.17 ± 0.03	**0.15 ± 0.05**
	ST_PREF	0.83 ± 0.01	**0.86 ± 0.01**	0.20 ± 0.03	**0.15 ± 0.04**
	POPT	0.77 ± 0.02	**0.80 ± 0.01**	0.28 ± 0.07	**0.18 ± 0.06**

The best-performing scores for each metric are highlighted in bold.

### 4.5 Inference time and GPU memory usage

We have quantified model capacity (number of trainable parameters), peak GPU memory usage during inference, and wall-clock inference time per subject across all 21 HCP test subjects for TractSeg, FODSeg_mc and FODSeg_sc, to contextualize the computational cost of the proposed models. The results are given in Table 7. All measurements were performed on the same GPU (NVIDIA RTX 4090) with batch size 1.

**Table 7:** Parameter counts, peak GPU memory usage, and approximate inference time per subject for TractSeg, FODSeg_mc (multi class), and FODSeg_sc (single class) on the HCP test set.

Method	Output type	Params/network (M)	Params across 72 tracts (M)	Peak GPU memory (GB)	Mean inference time per subject (seconds)
TractSeg	Multi-class, 72 tracts from peaks	37.04	37.04	0.71	27.5*
FODSeg_mc	Multi-class, 72 tracts from full fODF	37.06	37.06	0.52	3.64
FODSeg_sc	Single-class, 1 tract from full fODF	36.87	2662.33	0.38	2.89 (per tract)

*Runtime for TractSeg, we timed the full command-line interface call (TractSeg -i peaks.nii.gz), which includes internal pre- and post-processing steps and disk I/O (reading/writing multiple NIfTI volumes). FODSeg models work on numpy slices and have minimum overhead. As a result, the absolute inference times are not strictly comparable between TractSeg and FODSeg models.

For FODSeg_mc and FODSeg_sc, which are implemented in PyTorch, we instantiated the corresponding 2D U-Net architectures and counted parameters as the sum over all trainable tensors. Inference time and peak GPU memory were measured using a slice-wise inference script that processes all 21 HCP test subjects. For each subject, we performed 2D inference in the axial, sagittal and coronal orientations and then performed post-processing by averaging the three probability maps and thresholding at 0.5 to obtain the final 3D tract mask. Peak GPU memory was recorded during the same runs.

For TractSeg, we relied on the official implementation for pretrained weights. We obtained parameter count by loading the pretrained_weights_tract_segmentation_v3.npz checkpoint with Pytorch and summed over all tensors. To measure inference time, we executed a script to run tract segmentation (TractSeg -i < subject>peaks.nii.gz) for each of the 21 HCP test subjects and recorded the wall-clock time. Because TractSeg runs as a separate process, peak GPU memory was estimated using “nvidia-smi –query-gpu = memory.used” sampled once per second on an otherwise idle GPU; the maximum observed during tract segmentation was recorded, which we report as TractSeg’s peak GPU memory during inference.

## 5 Discussion

In this work, we proposed FODSeg, a voxel-wise segmentation framework that utilizes the full angular information from fODFs and reformulates white matter tract segmentation as a single-class problem. By modeling each tract independently and leveraging complete fODF representations, FODSeg improved segmentation accuracy, especially in anatomically complex regions where standard multi-class, peak-based methods such as TractSeg often struggle. We evaluated our model extensively across all 72 tracts in the HCP dataset, demonstrating robust overall performance, tract-wise accuracy, and improved precision in crossing fiber tracts and bottleneck regions. Below, we discuss the key findings in depth and implications of our results.

### 5.1 General discussion

The median Dice improvement of the proposed FODSeg over TractSeg was 0.014 with improvements ranging from −0.09 to 0.16. Wilcoxon signed-rank tests comparing Dice scores across all tracts show that FODSeg_mc significantly outperforms TractSeg (*p* < 0.001), and FODSeg_sc significantly outperforms both TractSeg (*p* < 0.001) and FODSeg_mc (*p* < 0.001). Although the overall average Dice improvement of FODSeg over TractSeg across all 72 tracts is modest (~0.015), this summary conceals important tract-level differences. When examined individually, FODSeg outperforms TractSeg in 50 of the 72 tracts, performs comparably in 7 tracts, and is surpassed by TractSeg in only 15 tracts. These results confirm that both full fODF input and single-class modeling contribute to consistent and statistically significant improvements in segmentation accuracy. Beyond statistical significance, these improvements have meaningful implications for both research and clinical applications. Enhanced segmentation accuracy, particularly in tracts with complex geometries, enables more precise structural connectivity analysis and downstream feature extraction. Moreover, the use of full fODF input preserves rich directional information often lost in peak-based representations, allowing the model to better disambiguate tracts with overlapping spatial trajectories. The consistent gains across a large number of tracts (over 70% showing improved Dice and reduced overreach) suggest that the proposed framework is robust across diverse anatomical configurations, from large projection tracts like the CST to smaller, curved tracts such as the UF. This broad generalizability highlights the adaptability of FODSeg_sc to varying tract shapes, sizes, and orientations.

### 5.2 Tract-wise improvement

As shown in Table 2, our method FODSeg_sc performs competitively across all tracts, frequently outperforming streamline or voxel-based methods such as Recobundles, TractSeg, U-Net, Classifyber and FODSeg_mc. For example, on the left ILF and right IFO, our method achieves the highest Dice scores (0.89 and 0.84, respectively), demonstrating robust segmentation of long-range association tracts that traverse regions of complex fiber architecture and partial volume effects.

Furthermore, our performance remains consistently strong across diverse tracts, offering a balanced trade-off between accuracy and generalizability. For example, previous work has shown that certain anatomically small and structurally complex tracts, such as the commissure anterior (CA) and fornix (FX), pose significant challenges for segmentation models due to their thin geometry, pronounced curvature and low signal-to-noise ratio (Wasserthal et al., 2018). FODSeg_sc demonstrates promising improvements in these areas. Specifically, our model achieves an average Dice score of 0.70 for CA and 0.77 for the left and right FX, compared to 0.68 and 0.69, respectively, using TractSeg. These results indicate robust performance despite these tracts being among the most difficult to delineate. These improvements suggest that leveraging the full angular information of the fODF and training tract-specific models enhances the model’s ability to resolve thin or less prominent tracts, where partial volume effects and noise can otherwise hinder segmentation accuracy.

### 5.3 Comparison with Classifyber

Classifyber (Berto et al., 2021) has recently been proposed as a streamline-based alternative for white matter tract segmentation, with evaluations reported on HCP-major, HCP-minor, HCP-IFOF, and a clinical dataset of epilepsy patients (Berto et al., 2021). Classifyber achieves higher Dice scores than FODSeg_sc for several association and limbic bundles. This is consistent with its formulation as a per-bundle binary classifier in streamline space where each model operates on a compact feature vector that encodes global and local streamline geometry, endpoint connectivity, and distances to anatomically defined ROIs, which appears particularly well suited to discriminating complex long-range trajectories. While the method has shown strong performance on selected bundles, it has not been evaluated against TractSeg across the full set of 72 white matter tracts. By contrast, our work systematically evaluates FODSeg on all 72 bundles, enabling a comprehensive tract-wise assessment. Notably, in the HCP-minor dataset, the Classifyber authors report that *“the mean improvement in terms of DSC with respect to the second-best method is 0.09”* (Berto et al., 2021) for 8 representative tracts. In comparison, FODSeg achieves average improvements ranging from 0.01 to 0.16 across 50 tracts, indicating that our gains, while sometimes smaller in magnitude, are widespread and consistent rather than concentrated in a small subset of bundles.

It is also important to highlight key differences in representation and test-time computation. Classifyber requires whole-brain tractograms as input and uses independent classifiers for each bundle to assign streamlines to tracts. At inference, each streamline must be evaluated against multiple bundle-specific classifiers, which increases computational demands when many bundles are considered and may result in individual streamlines being assigned to several bundles unless post-processing heuristics are applied. Moreover, pretrained Classifyber models are currently available only for a subset of tracts, and extending the approach to the full set of 72 bundles would require generating additional training tractograms and expert-curated bundle labels for each new tract. In contrast, FODSeg directly predicts voxel-wise segmentations from fODFs, avoiding the need for tractography preprocessing, scaling efficiently to all 72 bundles, and inherently sidestepping streamline overlap issues. Thus, while Classifyber demonstrates strong results in selected datasets, FODSeg offers a more systematic and scalable solution, providing consistent improvements across the full white matter.

### 5.4 Crossing fiber and bottleneck issues

White matter bottlenecks are anatomically complex regions where multiple tracts converge into overlapping spatial configurations, often sharing a single voxel orientation while maintaining distinct trajectories. These regions are particularly challenging for tract segmentation, as standard methods may struggle to distinguish between tightly interwoven tracts. Bottleneck areas have been shown to account for over 50%–70% of fixels in the human white matter, highlighting the critical need for precise segmentation approaches in these zones. To evaluate the robustness of FODSeg_sc, we compared it to TractSeg across a curated set of tracts known to pass through major bottleneck regions (occipital lobe, brainstem, and internal capsule). In addition, we also included results from the multi-class version (FODSeg_mc) to provide a fairer comparison. As shown in Table 3, our method achieved consistently improved or equivalent results across multiple evaluation metrics, particularly in terms of Volumetric Overreach (VOR), which quantifies false positive volume. Notably, FODSeg_mc produces the lowest VOR values in several tracts, demonstrating clear reductions in false positives compared to both TractSeg and FODSeg_sc. For example, VOR was reduced from 0.24 (TractSeg) to 0.13 (FODSeg_mc) in the OR, and from 0.31 (TractSeg) to 0.22 (FODSeg_mc) in CC_7. Reductions in VOR (TractSeg vs. FODSeg_sc) were observed across nearly all tracts, most notably in the OR (0.24 vs. 0.15), CC_7 (0.31 vs. 0.25), and CST (0.14 vs. 0.10), demonstrating that our approach is more conservative and precise in complex regions, reducing over-segmentation while maintaining accurate delineation. Furthermore, FODSeg_sc maintains or improves performance in Dice and Jaccard scores, indicating a higher degree of spatial agreement with the ground truth. For example, the ST_POSTC tract shows an increase in Dice (TractSeg vs. FODSeg) (0.85 vs. 0.91) and Jaccard (0.73 vs. 0.83), suggesting that our method captures the tract more completely without incorporating extraneous voxels. While FODSeg_mc also improves VOR for this tract (0.08), its Dice score (0.90) is slightly lower than FODSeg_sc (0.91), reflecting the trade-off between reducing false positives and preserving segmentation accuracy. Improvements in precision further reflect this trend, suggesting fewer false positives. Importantly, specificity remains consistently high (0.99) across both methods, reinforcing that neither method compromises background suppression. Overall, these results affirm that segmenting each tract individually from the full fODF, as performed in our method, offers significant advantages in anatomically ambiguous regions. While FODSeg_mc clearly reduces false positives, this often comes at the cost of slightly lower Dice scores. By contrast, FODSeg_sc provides the best balance between accuracy and reduction of false positives. By avoiding the multi-class prediction strategy of TractSeg and instead leveraging a dedicated single-class segmentation framework, our approach achieves better localization and tract-specific delineation, especially in regions prone to crossing or merging fibers.

### 5.5 Clinical quality data

The results in Table 4 demonstrate that FODSeg_sc maintains robust performance even when trained using single-shell (*b* = 1,000 s/mm^2^) CSD, which better approximates the acquisition protocols used in clinical diffusion MRI. Across representative tracts such as the CST and UF, FODSeg_sc achieved Dice scores nearly identical to TractSeg. For clinically relevant tracts including the AF, ILF, and IFO, FODSeg_sc consistently matched or slightly outperformed TractSeg, with the largest gains observed in the left AF (+0.02) and right ILF (+0.02). In bottleneck regions, which are particularly challenging due to the convergence of multiple tracts with similar orientations, FODSeg_sc also demonstrated competitive results. Improvements were observed (TractSeg vs. FODSeg) in the optic radiation (OR: 0.85 vs. 0.87), middle longitudinal fasciculus (MLF: 0.86 vs. 0.87), and superior thalamic radiation (STR: 0.79 vs. 0.80). Notably, FODSeg_sc achieved higher Dice in the ST_PREF tract (0.86 vs. 0.90), while maintaining comparable performance in other bottleneck tracts such as the CST and POPT. Overall, these findings suggest that the combination of full fODF input and single-class modeling provides advantages even under constrained single-shell acquisitions. This indicates that FODSeg is not strictly dependent on high-quality multi-shell data and can generalize to more limited acquisition settings, enhancing its potential clinical applicability. While the improvements are modest in some cases, the results highlight the robustness of the framework and its ability to mitigate bottleneck and crossing-fiber ambiguities under clinically realistic conditions.

To further evaluate robustness under clinically realistic acquisition conditions, we simulated a clinical-quality dataset by downsampling HCP data to 2.5 mm isotropic resolution and retaining only 32 directions at *b* = 1,000 s/mm^2^. This dataset mimics protocols commonly used in clinical practice, with reduced spatial and angular resolution compared to research-grade acquisitions. As summarized in Table 5, FODSeg_sc substantially outperforms TractSeg across all global evaluation metrics. Dice improved from 0.759 ± 0.06 (TractSeg) to 0.836 ± 0.05. Importantly, volumetric overreach (VOR), which quantifies false positives, was reduced by nearly half from 0.283 ± 0.19 (TractSeg) to 0.179 ± 0.16. Specificity remained near ceiling for both methods (0.998–0.999), indicating that gains are not attributable to background suppression.

Tract-wise analysis in Table 6 reveals that these improvements are consistent across both representative and bottleneck tracts. In bottleneck regions, reductions in VOR were particularly notable: from 0.38 to 0.09 in the right ILF, from 0.35 to 0.15 in the left ILF, and from 0.28 to 0.10 in the ST_POSTC. These results suggest that FODSeg_sc is better able to disambiguate overlapping orientations in complex regions, while also avoiding extraneous over-segmentation.

These findings demonstrate that the advantages of FODSeg are not limited to research-grade HCP acquisitions. Even when evaluated on a simulated clinical dataset with significantly degraded resolution and angular coverage, FODSeg_sc maintains strong performance and consistently outperforms TractSeg across both global and tract-specific metrics. This robustness highlights the translational potential of our framework, suggesting that improvements in segmentation accuracy and reductions in false positives may carry over to real-world clinical acquisitions where data quality is limited.

From a generalizability standpoint, these clinical-quality experiments highlight that FODSeg is primarily tied to the fODF/SH representation rather than to a fixed acquisition protocol. The networks used here operate on 45-channel fODFs corresponding to spherical harmonic coefficients up to *l*_max_ = 8, estimated with a multi-tissue CSD pipeline. In principle, any diffusion acquisition from which compatible fODFs can be computed (same SH order and basis, similar preprocessing) can be fed into the trained models, and our results on single-shell and downsampled data demonstrate that FODSeg_sc can retain good performance when retrained or fine-tuned on such target-domain data. At the same time, our findings also indicate that we should not expect complete out-of-the-box invariance to large domain shifts in *b*-values, angular resolution, or SH order. For acquisitions that differ substantially from the HCP-like setting, particularly those requiring a lower SH order, the safest practice is to adapt the input dimensionality and fine-tune or retrain the models on representative subjects. In contrast, for datasets where fODFs are estimated with *l*_max_ = 8 using a similar CSD pipeline, the existing FODSeg models can be applied directly, with the understanding that modest acquisition differences may still influence performance and warrant validation on a subset of the target population.

### 5.6 Architectural considerations

It is important to note that the novelty of FODSeg does not lie in proposing a new backbone architecture. Instead, our contribution is in reformulating how diffusion MRI data are represented and modeled, through the use of full fODFs and single-class training. While our framework employs a standard 2D U-Net for reproducibility and efficiency, we also explored alternative architectures. Specifically, we trained 3D U-Nets with both peak-based and full fODF inputs, which achieved lower Dice scores (0.7964 for peak-based model and 0.802 for full fODF-based model across all 72 white matter tracts) compared to our proposed framework. A possible explanation for these drops in accuracy is that training with 3D U-Nets substantially reduces the number of available training samples (since entire 3D volumes are used rather than large numbers of 2D slices) and simultaneously increases computational complexity due to 3D convolutions, which can limit optimization efficiency and generalization. We also tested a vision transformer (ViT) model (Dosovitskiy et al., 2020); however, despite ~544,000 2D slices, the effective training diversity was limited to 63 subjects, which proved insufficient for ViTs. Prior work shows that ViTs typically require much larger and more diverse datasets (hundreds of thousands to millions of independent samples), often necessitating extensive pretraining with large datasets or aggressive augmentation to converge effectively, owing to their weak inductive biases compared to CNNs (Steiner et al., 2021). In our experiments, the ViT model failed to converge and was computationally prohibitive to scale to all 72 tracts. These findings support our choice to retain a 2D U-Net backbone and to focus our innovations on data representation and tract-specific training, which directly address key segmentation challenges such as crossing fibers and bottlenecks.

### 5.7 Computational considerations and scalability

Although FODSeg_sc adopts a tract-wise formulation and therefore uses one network per tract, its per-network capacity and memory footprint are closely matched to the multiclass baselines. Each FODSeg_sc model essentially has the same number of parameters, and a similar peak GPU memory requirement. The measured parameter counts show that per-network capacity is similar for TractSeg, FODSeg_mc and each FODSeg_sc model. Within this shared U-Net backbone and dataset, the single class formulation yields higher Dice and precision and lower volumetric overreach than the multi-class formulation. However, we note that the overall capacity of FODSeg across 72 tracts is larger than that of a single multi-class network, and we do not claim that a sufficiently widened multi-class model could not partially close this gap. Our experiments show that for a fixed backbone and dataset, moving from peaks to full fODFs and from multi-class to single-class training yields consistent improvements, but this does not preclude future multi-class architectures with greater capacity from achieving comparable performance. The total parameter storage for the full set of 72 tracts is larger for FODSeg_sc, but the peak memory at inference time will remain the same, because only one single-class model needs to reside on the GPU at any given moment.

In terms of runtime, FODSeg_sc running all 72 tract-specific models sequentially is slower than FODSeg_mc and TractSeg, when the full tract set is needed. However, FODSeg_sc offers two practical advantages. First, many applications focus on a small subset of tracts, in which case only the corresponding single-class models need to be evaluated, keeping inference times comparable to or faster than multi-class approaches. Second, the tract-wise formulation allows parallel evaluation across tracts on multi-GPU systems or clusters, so wall-clock time can be substantially reduced in settings where parallel resources are available.

Overall, these results indicate that the proposed single-class FODSeg architecture remains computationally tractable in practice while providing the best trade-off between accuracy and reduced overreach, especially when attention is restricted to anatomically or clinically critical tracts.

### 5.8 Impact of FODSeg

The improvements in segmentation precision of FODSeg, in both crossing fiber tracts and in bottleneck regions, have important implications beyond general accuracy metrics. In neurosurgical planning, precise delineation of white matter tracts is crucial for avoiding damage to eloquent pathways, such as the corticospinal tract or optic radiation, which are often embedded in densely intersecting regions (Zhang et al., 2022). Even small reductions in false positives or spatial overreach can increase surgeon confidence in patient-specific tractography maps, contributing to safer resection margins and better outcomes (Essayed et al., 2017; Zhang et al., 2022). From a research and translational standpoint, enhanced segmentation accuracy improves the anatomical validity of tract-specific connectivity measures used in structural connectivity studies or tractometry pipelines, for example, along-tract fractional anisotropy (Chandio et al., 2020) or apparent fiber density profiling (Raffelt et al., 2012), which are increasingly employed in neurodevelopmental (Kojima et al., 2024) and neuropsychiatric studies (Wasserthal et al., 2020). Although FODSeg outputs only binary voxel-wise tract masks, these can be directly combined with existing tractography and fixel-based workflows. Tract-specific tractograms can be generated by seeding streamlines inside a FODSeg mask and constraining them to remain within the mask, and the resulting streamlines can be used for standard along-tract analysis. For fixel-based analysis, the same masks can be intersected with fixel maps to select fixels for each bundle, optionally refined by enforcing consistency between fixel orientations and tract-specific streamlines. In this sense, FODSeg acts as a flexible masking and selection tool that provides anatomically informed constraints for downstream streamline- and fixel-based quantitative analyses, rather than replacing those steps.

Furthermore, the statistical power of clinical trials or cohort studies that use tract-based biomarkers can be substantially impacted by segmentation quality. As previously noted in simulation studies (Schilling et al., 2022), even modest gains in Dice or reduction in overreach translate into reduced measurement variance/error, which can lower the required sample size to detect group differences or treatment effects. Thus, the gains observed with FODSeg over TractSeg are not merely technical but may have real-world downstream benefits, especially in studies with limited sample sizes or in vulnerable populations such as infants or patients with pathology-distorted anatomy (Calixto et al., 2025). However, our current models are trained solely on anatomically typical adult HCP data, and recent work has shown that deep-learning-based segmentation can exhibit failure modes in the presence of severe anatomical distortion (Gruen et al., 2025). Extending and validating FODSeg in infants, patients with pathology-distorted anatomy, or other clinically heterogeneous populations will therefore require dedicated training data and careful evaluation. Also, while reductions in volumetric overreach relative to these expert-corrected groundtruth masks suggest improved spatial specificity, definitive conclusions about clinical impact will require validation against more direct anatomical ground truths (e.g., intra-operative mapping by electrostimulation during awake surgery or histological studies), which are beyond the scope of this work.

While we have not directly validated these downstream applications in the present work, they represent an important next step for future research. More broadly, these considerations highlight the general impact of having an accurate tract segmentation model: the improvements demonstrated with FODSeg over TractSeg may translate into real-world benefits for both clinical and research applications.

### 5.9 Future work

Future work will focus on extending FODSeg to downstream applications such as neurosurgical planning, tractometry, or connectivity analyses. The tract-wise improvements and reduction in false positives demonstrated here suggest that FODSeg could enhance the accuracy and interpretability of these analyses, which we aim to investigate in follow-up work. In addition, we aim to evaluate the performance of FODSeg in pathological populations, such as individuals with white matter abnormalities or neurodevelopmental disorders, where structural deviations from typical anatomy can affect segmentation accuracy.

Furthermore, we plan to extend our approach to fetal and neonatal white matter tract segmentation, a setting where white matter architecture differs substantially from that of adults. Neonatal tracts often vary in location, maturity, and even existence, making generalization from adult-trained, multi-class models unreliable. By training single-class segmentation models, FODSeg offers tract-specific adaptability that may enable better transferability to fetal/neonatal data. This flexibility is especially important for investigating early brain development and identifying biomarkers for neurodevelopmental risk. Finally, future work may also explore incorporating uncertainty estimation and confidence-aware predictions, which could enhance interpretability and clinical trust in tract segmentation results.

## 6 Conclusion

In this work, we present FODSeg, a voxel-based segmentation method that leverages a standard UNet architecture to directly segment white matter tracts from fiber orientation distributions (fODFs). Unlike traditional approaches that rely on intermediate tractography or streamline classification, FODSeg operates directly on fODFs estimated via constrained spherical deconvolution, enabling efficient and anatomically precise segmentation without the need for whole brain tractograms. We demonstrated the effectiveness of our method through extensive quantitative evaluations across all 72 tracts in the HCP dataset, tract-specific benchmarks, and challenging white matter bottleneck regions. FODSeg consistently outperformed voxel-wise baselines such as TractSeg and U-Net and performed competitively with streamline-based methods, while maintaining the advantages of full automation and voxel-level resolution. Our results highlight the strength of learning from fODFs in the spherical domain and showcase the potential of FODSeg for robust, scalable, and interpretable white matter segmentation in both research and clinical settings.

## Data Availability

The original contributions presented in the study are included in the article/Supplementary material, further inquiries can be directed to the corresponding authors.

## Footnotes

1
https://zenodo.org/records/1477956

2
https://github.com/ankitajoshi15/FODSeg

3
https://github.com/MIC-DKFZ/TractSeg
