# Supplementary material for: FODSeg: a deep learning framework for tract-specific white matter segmentation from full angular distributions
Source: Front Neurosci. 2026 Jan 12;19:1734498. doi: 10.3389/fnins.2025.1734498 (PMC12832898; doi:10.3389/fnins.2025.1734498)
Supplement: Supplementary file 1 [file Data_Sheet_1.docx]

Table 1: Summary of the evaluation metrics used to assess results

| **Metric** | **Description** | **Formulation** | **Usage** |
| --- | --- | --- | --- |
| Dice Score | Measures the overlap between the predicted segmentation and the ground truth. | $Dice= \frac{2 \vert Predicted \cap Groundtruth\vert}{\left\vert Predicted \right\vert+\vert Groundtruth\vert}$ | For balanced overlap;  score ranges from 0 to 1, where 1 indicates perfect overlap |
| Volumetric Overlap (VOP) | Quantifies the proportion of ground truth voxels that are correctly predicted | $VOP= \frac{\vert Predicted \cap Groundtruth\vert}{\vert Groundtruth\vert}$ | For under-segmentation / recall |
| Volumetric Overreach (VOR) | Evaluates the proportion of the predicted tract volume that falls outside the ground truth | $VOR= \frac{\vert Predicted \backslash Groundtruth\vert}{\vert Predicted\vert}$ | For over-segmentation / false positives; lower VOR indicates fewer false positives and better spatial precision |
| Specificity | Measures the proportion of true negative voxels that are correctly identified | $Specificity= \frac{\vert True Negatives\vert}{\vert True Negatives\vert+\vert False Positives\vert}$ | For avoiding false positives in background |
| Precision | Measures the fraction of predicted positive voxels that are correctly identified | $VOP= \frac{\vert Predicted \cap Groundtruth\vert}{\vert Predicted\vert}$ | Closely related to VOR  $VOR = 1-Precision$ |
| Jaccard Index | Quantifies the intersection-over-union between prediction and ground truth | $Jaccard= \frac{\vert Predicted \cap Groundtruth\vert}{\vert Predicted \cup Groundtruth\vert}$ | Closely related to Dice  $Dice = \frac{2. Jaccard}{1+Jaccard}$ |

Table 2: Quantitative comparison of segmentation performance for selected tracts passing through bottleneck regions. The results are shown for TractSeg and the proposed FODSeg single class segmentation (FODSeg_sc) method across six evaluation metrics. Tracts were selected due to their anatomical complexity and prevalence in major bottleneck regions of the white matter, where accurate segmentation is particularly challenging.

| **Bottleneck Region** | **Tract** | **Specificity↑** | | **Precision↑** | | **Jaccard↑** | |
| --- | --- | --- | --- | --- | --- | --- | --- |
|  |  | TractSeg | FODSeg | TractSeg | FODSeg | TractSeg | FODSeg |
| Occipital (anterior - posterior) | CC_7 | 0.99 | 0.99 | 0.74 | 0.77 | 0.64 | 0.67 |
|  | OR | 0.99 | 0.99 | 0.78 | 0.85 | 0.68 | 0.75 |
|  | MLF | 0.99 | 0.99 | 0.83 | 0.85 | 0.75 | 0.76 |
|  | IFO | 0.99 | 0.99 | 0.78 | 0.80 | 0.68 | 0.69 |
| Brainstem (superior - inferior) | T_PREM | 0.99 | 0.99 | 0.84 | 0.86 | 0.72 | 0.75 |
|  | STR | 0.99 | 0.99 | 0.79 | 0.77 | 0.67 | 0.67 |
|  | ST_POSTC | 0.99 | 0.99 | 0.84 | 0.90 | 0.73 | 0.83 |
| Internal capsule (superior -inferior) | CST | 0.99 | 0.99 | 0.85 | 0.85 | 0.74 | 0.75 |
|  | ST_PREF | 0.99 | 0.99 | 0.87 | 0.85 | 0.80 | 0.77 |
|  | POPT | 0.99 | 0.99 | 0.81 | 0.81 | 0.72 | 0.71 |
